# The cross correlation properties of composite systems

**DOI:** 10.1038/s41598-017-18135-x

**Published:** 2018-01-22

**Authors:** Zhifu Huang, Shuqing Zheng

**Affiliations:** College of Information Science and Engineering, Huaqiao University, Xiamen, 361021 People’s Republic of China

## Abstract

A new method is presented for characterizing cross correlations in composite systems described by a couple of time-dependent random variables. This method is based on (i) rescaling the time derivatives of the variables to make their variances unity and then (ii) recombining these rescaled variables into their sum and difference. This manipulation enables one to express the joint probability distribution function in a peculiar way. It is also found that the entropy of composite systems is not equal to the sum of entropy of each subsystem because of the cross correlations.

## Introduction

The relationship of the whole and its parts is an important theme in science. In research, the whole can be regarded as composite systems of its parts. When the interaction of different parts is infirm, the cross correlation properties of different parts do no exist. However, when the interaction of different parts can not be ignored, the cross correlation properties of composite systems may be unknown. First of all, to obtain the cross correlation properties of the whole and its parts, the joint probability distribution function (JPDF) of the real composite systems should be analysed. The key to the research is to calculate the expression of JPDF from the obtained data^1^.

The properties of generalizing statistical mechanics through the nonadditive entropy were proposed in 1988^2^, namely ${S}_{q}=k\mathrm{(1}-{\sum }_{i=1}^{W}{p}_{i})/(q-\mathrm{1)}$, it reveals the additive entropy when *q* → 1. In addition, through the maximum entropy principle *δ*(*S*_*q*_ − *α*∑_*i*_*p*_*i*_ − *βU*) = 0, one can get the *q*-Gaussian distribution^2,3^. During the past three decades, this theory has been widely researched (see the Bibliography in http://tsallis.cat.cbpf.br/biblio.htm), especially promote the development of verifications, applications and predictions in physical systems and other scientific fields. In addition, when they researched the composite systems, the JPDF usually be verified as independent of its parts^2–4^. However, the JPDF of composite systems having cross correlations may not suit the independent principle of multiplying, it is still an opening problem in statistical physics^1,5,6^.

In the previous work, we introduced a unified way to acquire the JPDF in a single complex systems having long-term memory^7^. The JPDF is about two different events that occur at two time intervals. Since JPDF can be obtained from a single complex system, we can try to research the JPDF of composite systems. Firstly, we focus on the probability distribution function (PDF) of each subsystem. The concept of central limit theorem is of great importance in the theory of probability and also be crucial to statistical physics^1–8^. Basically, the sum of *N* independent identically distributed random variables, rescaled with a factor $1/\sqrt{N}$, agrees with the Gaussian distribution. However, the PDFs of variables in complex systems usually do not suit the form of Gaussian distribution due to the nearly independent or independent interactions, and suit the non-Gaussian distribution, for example L*é*vy stable form^9–12^, or the form of *q*-Gaussian given by

1 $$p(y)=C{\mathrm{[1}-\mathrm{(1}-q)\beta {y}^{2}]}^{\mathrm{1/(1}-q)},$$

where *q* and *β* are parameters characterizing the distribution^2,3^, while *C* is the normalized parameter. Here, *q* ≠ 1 means the form deviates the Gaussian shape, when *q* tends to 1, it converges to the Gaussian distribution. In recent researches, the *q*-Gaussian distribution is gaining attention from many scientific branches. For example, Caruso *et al*.^13^. observed that the probability distribution of energy differences of subsequent earthquakes in the World Catalog and Northern California is well fitted by a *q*-Gaussian with *q* = 1.75. Cai *et al*.^14^ found that the PDF of the detrended electroencephalogram signals is well fitted by a *q*-Gaussian distribution. In addition, DeVoe^15^ obtained a *q*-Gaussian distribution in a trapped ion interacting with a classical buffer gas. It was also found in turbulence experiments by Combe *et al*.^16^ that a *q*-Gaussian distribution is fitted well in the probability density function of the displacement fluctuations. Therefore, in the present work, we will try to compute the *q* value by fitting the *q*-Gaussian distribution.

When *q* > 1 and ${y}^{2}\ll \mathrm{1/[(}q-\mathrm{1)}\beta ]$, equation (1) will be converted as *p*(*y*) ∝ *y*^*γ*^, which is the power-law shape, here *γ* = 2/(1 − *q*). Thus we can assume that the asymptotic of L*é*vy stable forms can be investigated by the PDF in the *q*-Gaussian shape^17^. Since the distribution function must be normalized ${\int }_{-\infty }^{+\infty }p(y)dy=1$, one can obtain the normalized parameter *C* depends on *β* and *q* as

2 $$C=\frac{\sqrt{\beta (q-\mathrm{1)}}{\rm{\Gamma }}\mathrm{[1/(}q-\mathrm{1)]}}{\sqrt{\pi }{\rm{\Gamma }}\mathrm{[(3}-q\mathrm{)/(2}q-\mathrm{2)]}},$$

where Γ(...) is the gamma function, for 1 < *q* < 3. After that, we provide the variance ${\sigma }^{2}={\int }_{-\infty }^{+\infty }{y}^{2}p(y)dy$ of equation (1), which also depends on *β* and *q* as

3 $${\sigma }^{2}=\frac{{\rm{\Gamma }}\mathrm{[(5}-3q\mathrm{)/(2}q-\mathrm{2)]}}{2\beta (q-\mathrm{1)}{\rm{\Gamma }}\mathrm{[(3}-q\mathrm{)/(2}q-\mathrm{2)]}},$$

where 1 < *q* < 1.6. From equations (2) and (3), we can find that the *C* and *σ* all depend on the *β* and *q*. In another word, only two parameters are independent among *β*, *q*, *C*, and *σ*. Thus one can research the PDF by arbitrary two among them. Here we will choose *q* and *σ* to analyse the PDF of velocity in the system.

**Figure 1:**
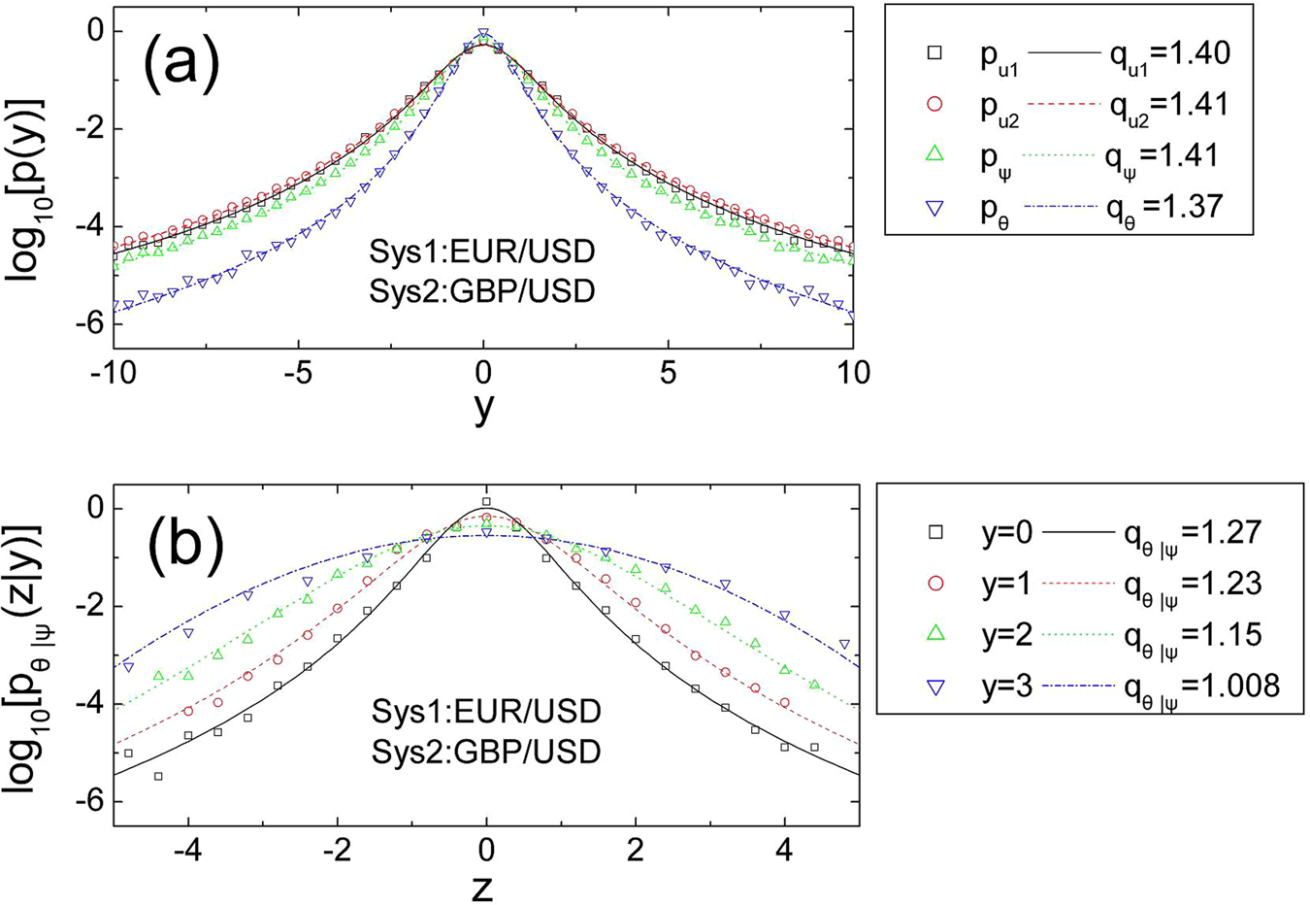
The PDFs of variables and the CPDFs between *θ*(*t*, Δ*t*) and *ψ*(*t*, Δ*t*).

Financial markets are becoming a paradigm of complex systems^18–22^. It is well documented about Borlands previous research^21^ that the data of NASDAQ returns on the order of minutes fits the *q*-Gaussian distribution, with *q* = 1.43. In our work, we choose the currency exchange databases for analysing. We use three currency exchange databases from the websites: http://www.metaquotes.net and http://finance.yahoo.com, about Euro. vs. U.S. Dollar (EUR/USD), Great Britain Sterling Pound vs. U.S. Dollar (GBP/USD), and Australian Dollar vs. U.S. Dollar (AUD/USD). The data samples contain the opening exchange prices of from 1999.1.1 to 2014.12.31 on the order of minutes. In the actual systems, the time interval of arbitrary processes is limited. We first define the logarithmic form^18–21^ of the exchange price in the financial system as the position in physics as *R*(*t*) = *Log*[*price*(*t*)]. Thus, the expression of displacement is straightforward as *x*(*t*, Δ*t*) = *R*(*t* + Δ*t*) − *R*(*t*), and the corresponding velocity can be obtained as *v*(*t*, Δ*t*) = *x*(*t*, Δ*t*)/Δ*t*. Here Δ*t* is the time interval. We simply denote Δ*t* as 1. After that, the corresponding normalized velocity is *u*(*t*, Δ*t*) = *v*(*t*, Δ*t*)/*σ*_*v*_, where *σ*_*v*_ is the standard deviation of velocity. We must mention that the displacement in our work is usually referred as return^18–21^. Figure 1a shows that the *q*-Gaussian distribution can be well approximated by the PDF of variables from the data, while different cases have different values of *q*.

In order to describe the cross correlations of composite systems, we can research the normalized velocity of each subsystem, while *u*_1_ and *u*_2_ are the normalized velocity of the first and second subsystem respectively. After that, we can also define two variables as *ψ*(*t*, Δ*t*) = [*u*_1_(*t*, Δ*t*) + *u*_2_(*t*, Δ*t*)]/2 and *θ*(*t*, Δ*t*) = [*u*_2_(*t*, Δ*t*) − *u*_1_(*t*, Δ*t*)]/2. For the sake of convenience, variable *A*(*t*, Δ*t*) is simplified as *A* in expression. For example, may be, *u*_1_(*t*, Δ*t*), *u*_2_(*t*, Δ*t*), *ψ*(*t*, Δ*t*) and *θ*(*t*, Δ*t*) respectively, simplified as *u*_1_, *u*_2_, *ψ* and *θ*. We must mention here that the cross correlation properties depend on the interactions of composite systems. Although the interactions’ form of composite systems may be very complex, we can find that the variables *ψ*(*t*, Δ*t*) and *θ*(*t*, Δ*t*) include the normalized velocity from each system and may be used to analyse the cross correlations of composite systems. As a result, we may obtain cross correlation properties of composite systems from analysing the relationship of variables *ψ*(*t*, Δ*t*) and *θ*(*t*, Δ*t*). From the defining of *ψ*(*t*, Δ*t*) and *θ*(*t*, Δ*t*), one can find that when the events *u*_1_(*t*, Δ*t*) and *u*_2_(*t*, Δ*t*) are ensured, the events *ψ*(*t*, Δ*t*) = [*u*_1_(*t*, Δ*t*) + *u*_2_(*t*, Δ*t*)]/2 and *θ*(*t*, Δ*t*) = [*u*_2_(*t*, Δ*t*) − *u*_1_(*t*, Δ*t*)]/2 are also ensured at the same time. As a result, we can obtain that

4 $${p}_{{u}_{1},{u}_{2}}(y,z)=|J|{p}_{\psi ,\theta }(\frac{z+y}{2},\frac{z-y}{2})$$

where |*J*| is the determinant of the Jacobian and equals 1/2, and ${p}_{{u}_{1},{u}_{2}}(y,z)$ is the JPDF of events *u*_1_(*t*, Δ*t*) and *u*_2_(*t*, Δ*t*), and *p*_*ψ*,*θ*_[(*z* + *y*)/2, (*z* − *y*)/2] is the JPDF of events *ψ*(*t*, Δ*t*) = (*z* + *y*)/2 and *θ*(*t*, Δ*t*) = (*z* − *y*)/2. The JPDF ${p}_{{u}_{1},{u}_{2}}(y,z)$ and *p*_*ψ*,*θ*_[*y*, *z*] can be also given as ${p}_{{u}_{1},{u}_{2}}(y,z)={p}_{{u}_{1}}(y){p}_{{u}_{2}|{u}_{1}}(z|y)$ and *p*_*ψ*,*θ*_(*y*, *z*) = *p*_*ψ*_(*y*)*p*_*θ*|*ψ*_(*z*|*y*), where ${p}_{{u}_{1}}(y)$ is the PDF of event *u*_1_(*t*, Δ*t*) and *p*_*ψ*_(*y*) is the PDF of event *ψ*(*t*, Δ*t*). While ${p}_{{u}_{2}|{u}_{1}}(z|y)$ is the conditional PDF(CPDF) of event *u*_2_(*t*, Δ*t*) with respect to event *u*_1_(*t*, Δ*t*), and *p*_*θ*|*ψ*_(*z*|*y*) is the CPDF of event *θ*(*t*, Δ*t*) with respect to event *ψ*(*t*, Δ*t*). Thus, we can derive

5 $${p}_{{u}_{2}|{u}_{1}}(z|y)=\frac{1}{2}{p}_{\psi }(\frac{z+y}{2}){p}_{\theta |\psi }(\frac{z-y}{2}|\frac{z+y}{2})/{p}_{{u}_{1}(y)}$$

From equation (5), we can calculate the CPDF ${p}_{{u}_{2}|{u}_{1}}(z|y)$ from the CPDF *p*_*θ*|*ψ*_(*z*|*y*). Fortunately, due to the velocity in two systems have been normalized, the covariance of the *ψ*(*t*, Δ*t*) and the *θ*(*t*, Δ*t*) as $\langle \psi (t,{\rm{\Delta }}t)\theta (t,{\rm{\Delta }}t)\rangle =[\langle {u}_{2}^{2}(t,{\rm{\Delta }}t)\rangle -\langle {u}_{1}^{2}(t,{\rm{\Delta }}t)\rangle \mathrm{]/4}$ is equal to zero. It demonstrates that the correlation of *ψ*(*t*, Δ*t*) and *θ*(*t*, Δ*t*) is neither negative nor positive. Accordingly, we suppose the CPDF *p*_*θ*|*ψ*_(*z*|*y*) is a symmetrical mathematical function. Since the cross correlations of composite systems, the covariance of *u*_1_(*t*, Δ*t*) and *u*_2_(*t*, Δ*t*) may be not equal to 0. As a consequence, we can generate the asymmetric CPDF ${p}_{{u}_{2}|{u}_{1}}(z|y)$ from the symmetrical CPDF *p*_*θ*|*ψ*_(*z*|*y*) in composite systems. We can study two time series at the same moment. After that, two time series can be seen as composite systems. As we noted before, the covariance of *ψ*(*t*, Δ*t*) and *θ*(*t*, Δ*t*) is equivalent to zero, the CPDF *p*_*θ*|*ψ*_(*z*|*y*) is a symmetrical mathematical function. The CPDF *p*_*θ*|*ψ*_(*z*|*y*) can be written in the form of *q*-Gaussian distribution as

6 $${p}_{\theta |\psi }(z|y)={C}_{\theta |\psi }(y\mathrm{)\{1}-\mathrm{[1}-{q}_{\theta |\psi }(y)]{\beta }_{\theta |\psi }(y){z}^{2}{\}}^{\mathrm{1/[1}-{q}_{\theta |\psi }(y)]}$$

It is important to stress that the CPDF *p*_*θ*|*ψ*_(*z*|*y*) depends on *ψ*(*t*, Δ*t*) means the cross correlation in composite systems. Thus, the conditional parameters *C*_*θ*|*ψ*_(*y*), *q*_*θ*|*ψ*_(*y*) and *β*_*θ*|*ψ*_(*y*) all depend on *ψ*(*t*, Δ*t*). In the present work we show that there have no more than two independent parameters in the system, and investigate the conditional variance ${\sigma }_{\theta |\psi }^{2}(y)$ and the conditional *q*_*θ*|*ψ*_(*y*) which characterizes the conditional fat-tail of the distribution to obtain the expression of the CPDF *p*_*θ*|*ψ*_(*z*|*y*). Figure 1b shows the good fitting of the CPDF *p*_*θ*|*ψ*_(*z*|*y*) of the symmetrical *q*-Gaussian distribution shape. The values of *q* changes along with the change of the values of *y*. Accordingly, we draw the conclusion that the *q*-Gaussian distribution can be used to analyse the CPDF *p*_*θ*|*ψ*_(*z*|*y*) in composite systems.

**Figure 2:**
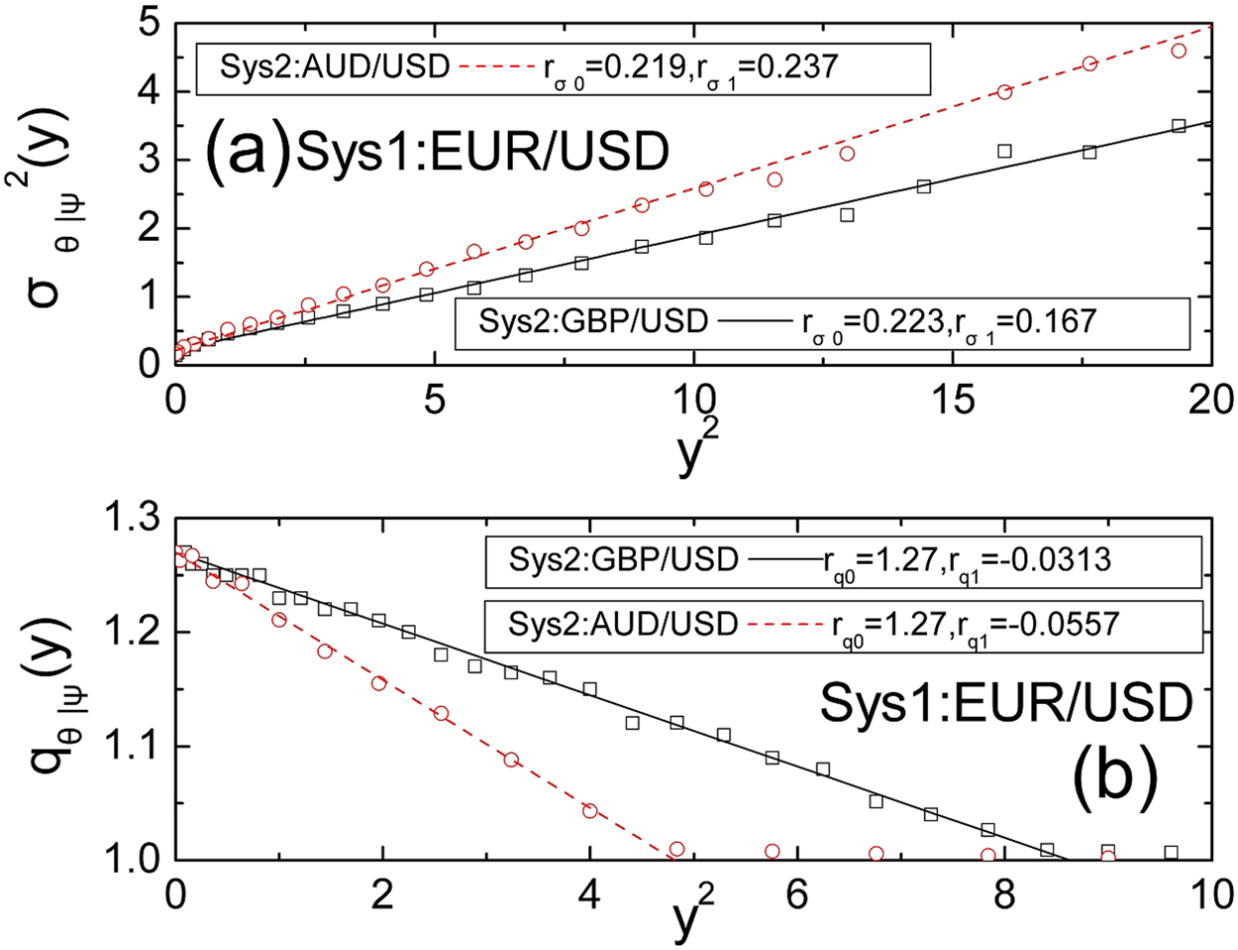
The conditional variance and the conditional *q*.

Because composite systems sometimes exhibit cross correlations, the relation of CPDF between the sum and the difference of normalized velocity will not be directly researched. Nevertheless, as the covariance of *ψ*(*t*, Δ*t*) and *θ*(*t*, Δ*t*) equals zero, the conditional variance ${\sigma }_{\theta |\psi }^{2}(y)$ is also a symmetrical function depends on *ψ*(*t*, Δ*t*). On the one hand, the conditional variance ${\sigma }_{\theta |\psi }^{2}(y)$ and [*ψ*(*t*, Δ*t*)]^2^ are both including the dimension of square velocity. On the other hand, because the conditional variance ${\sigma }_{\theta |\psi }^{2}(y)$ is an average value, when the [*ψ*(*t*, Δ*t*)]^2^ is large enough, the conditional variance ${\sigma }_{\theta |\psi }^{2}(y)$ will be not very larger than the [*ψ*(*t*, Δ*t*)]^2^. Based on these reasons, we construct the quadratic representation of the conditional variance ${\sigma }_{\theta |\psi }^{2}(y)$ as

7 $${\sigma }_{\theta |\psi }^{2}(y)={r}_{\sigma 0}+{r}_{\sigma 1}\,{y}^{2}$$

where *r*_*σ*0_ and *r*_*σ*1_ are the parameters of function ${\sigma }_{\theta |\psi }^{2}(y)$. Therefore, we can fit the conditional variance with equation (7). As can be shown in Fig. 2a, equation (7) can be well fitted by the data. It can also be seen that different composite systems may exist different conditional variances in Fig. 2a. It means that the cross correlation properties in different composite systems are not the same.

**Figure 3:**
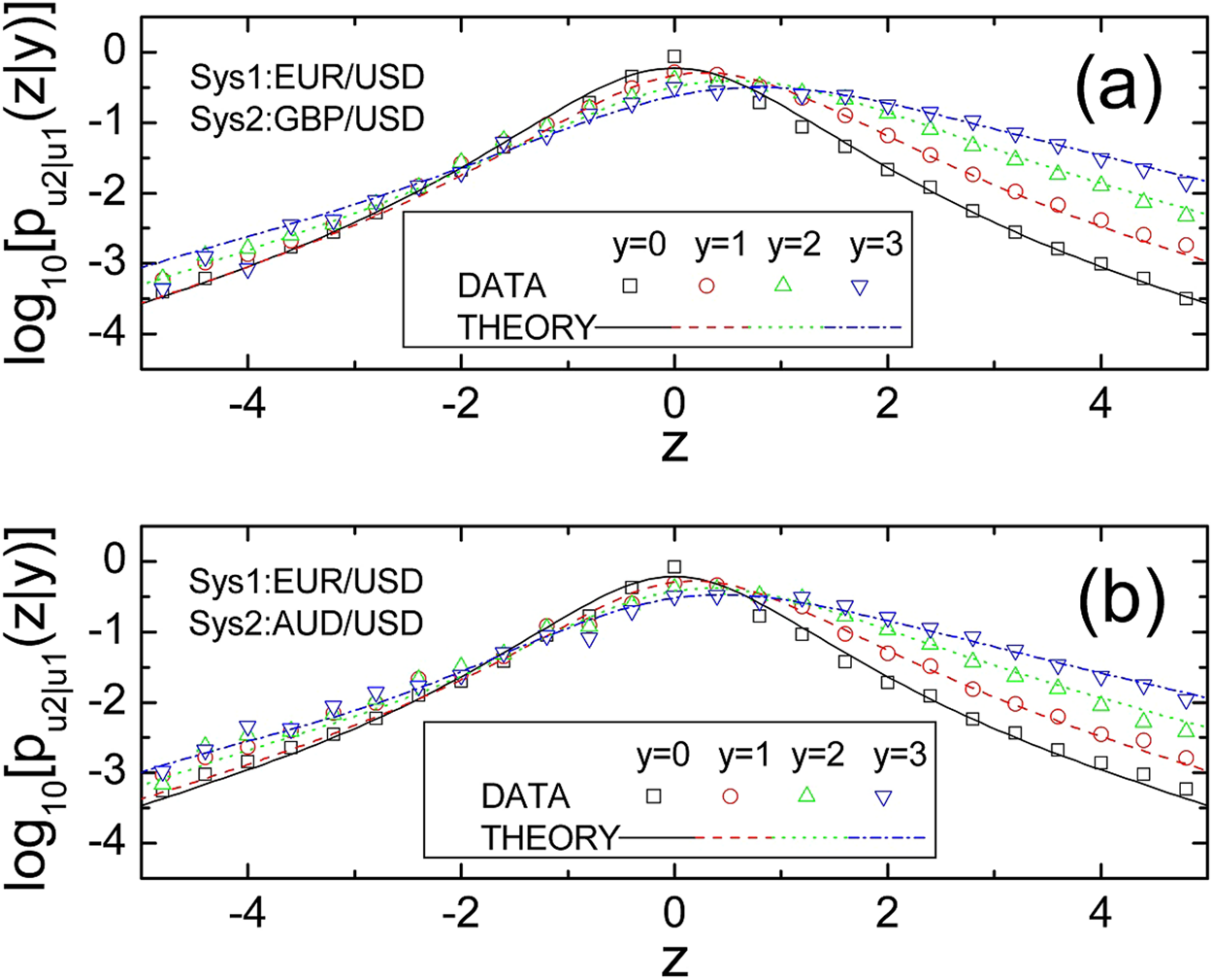
The CPDFs of the velocity in composite systems, (**a**) EUR/USD joint GBP/USD, (**b**) EUR/USD joint AUD/USD. The curves and dots respectively represent the real data and theory.

In addition, we can analyse the conditional *q* of the difference of normalized velocity versus the sum of normalized velocity. As the covariance of *ψ*(*t*, Δ*t*) and *θ*(*t*, Δ*t*) equals zero, the conditional *q* may depend on the square of the sum of normalized velocity. It is important to stress that with the increasing value of the sum of normalized velocity, the difference of normalized velocity is decreases frequently, and the corresponding conditional difference of normalized velocity will be independent of each other. Therefore, the conditional *q* will approach to 1 in the case of the sum of normalized velocity is large enough. As a result, we can adopt the value of conditional *q* equal to 1, only when the value of conditional *q* is less than 1.01. On this basis, we do not need to analyze the value of conditional *q* in the scope (1, 1.01). It indicates that the range of the conditional *q* is not very large. Thus, the conditional *q* values can be fitted to the following quadratic shape,

8 $${q}_{\theta |\psi }(y)={r}_{q0}+{r}_{q1}\,{y}^{2}$$

where *r*_*q*0_ and *r*_*q*1_ are two parameters of the function *p*_*θ*|*ψ*_(*z*|*y*). Figure 2b shows the good fitting of that equation (8) to the real data. It shows again that different composite systems exhibit different cross correlation properties. In a word, we can use the *q*-Gaussian distribution, conditional variance and conditional *q* value to calculate the CPDF *p*_*θ*|*ψ*_(*z*|*y*). Moreover, we can derive the expression of the CPDF *p*_*θ*|*ψ*_(*z*|*y*) by substituting equations (2), (3), (7) and (8) into equation (6), and we can generate the CPDF ${p}_{{u}_{2}|{u}_{1}}(z|y)$ and JPDF ${p}_{{u}_{1},{u}_{2}}(y,z)$ by equation (5). Consequently, we draw the conclusion that in any cases the CPDF ${p}_{{u}_{2}|{u}_{1}}(z|y)$ and JPDF ${p}_{{u}_{1},{u}_{2}}(y,z)$ can be explicitly obtained by the six parameters *q*_*ψ*_, *σ*_*ψ*_, *r*_*σ*0_, *r*_*σ*1_, *r*_*q*0_ and *r*_*q*1_, which can be obtained from the data fitting. According to Fig. 3, in different conditions, the theoretical curves can be well fitted by the data, and the function of the CPDF ${p}_{{u}_{2}|{u}_{1}}(z|y)$ is asymmetric when *y* ≠ 0. It is implied that the composite systems having cross correlations and their JPDFs do not suit the independent principle of multiplying. It is essential to illustrate that the expression of JPDF can be generalized to other composite systems, one can construct it in the same way.

**Figure 4:**
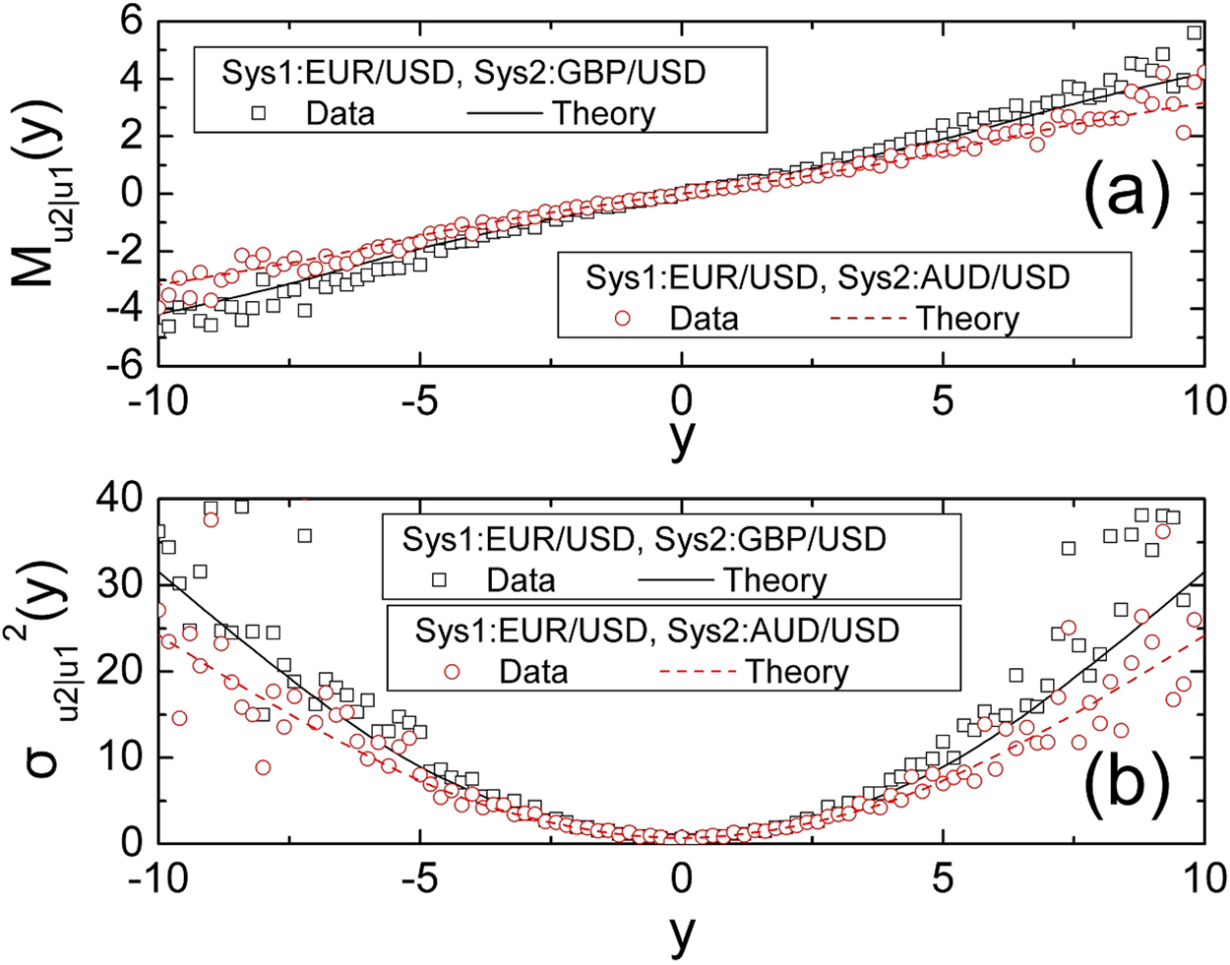
The conditional average of the normalized velocity and the conditional variance of the normalized velocity. Square dots and solid curves respectively represent the cases of real data and theory.

Furthermore, we may provide the conditional expectation of normalized velocity of composite systems by the obtained CPDF ${p}_{{u}_{2}|{u}_{1}}(z|y)$ as

9 $${M}_{{u}_{2}|{u}_{1}}(y)={\int }_{{W}_{z}|y}z{p}_{{u}_{2}|{u}_{1}}(z|y)dz$$

where ${M}_{{u}_{2}|{u}_{1}}(y)$ is the conditional average which depends on *u*_1_(*t*, Δ*t*), while *W*_*z*|*y*_ means the condition *u*_1_(*t*, Δ*t*) = *y* in all cases. It can be seen in Fig. 4a that the curves of the theory can be directly compared with empirical data. We can found that due to the cross correlation exists between composite systems, the conditional expectation tends monotonic, it means the mean velocity in the other system depends strongly on the mean velocity in the first system. We may also found that different composite systems exhibit different cross correlation properties, it is necessary to analyse the variables of composite systems. It can be seen from the Fig. 4a that the conditional average of EUR/USD joint GBP/USD is more than EUR/USD joint AUD/USD, it may mean that the cross correlation of EUR/USD joint GBP/USD is larger than EUR/USD joint AUD/USD.

Similarly, we can analogously construct the conditional variance of normalized velocity between composite systems as

10 $${\sigma }_{{u}_{2}|{u}_{1}}^{2}(y)={\int }_{{W}_{z}|y}{z}^{2}{p}_{{u}_{2}|{u}_{1}}(z|y)dz$$

Figure 4b shows the approximation of the theoretical curve with empirical data. It reveals the result of the cross correlation: with the small or large velocity in the first system, the conditional variance in the other systems may be small or large too. We must stress that the CPDF obtained before is the foundation of the research of the conditional variance and the conditional average, and the CPDF can lead to exploring other cross correlation properties of composite systems. Figure 4b also shows that the conditional variance of EUR/USD joint GBP/USD is more than EUR/USD joint AUD/USD, it means once again that the cross correlation of EUR/USD joint GBP/USD is larger than EUR/USD joint AUD/USD.

In addition, we can also analyse the nonadditivity of the entropy of composite systems based on the obtained JPDF. We can firstly numerically calculate the entropy of each subsystem by Shannon method^23^ as

11 $${S}_{u}=-k{\int }_{W}{p}_{u}(y)\,{ln}\,[{p}_{u}(y)]dy$$

where *k* is the Boltzmann parameter, for the sake of convenience, we set *k* equals 1 in our work. From the currency exchange databases, we can obtain that the entropy of EUR/USD, GBP/USD and AUD/USD equal 1.335, 1.328 and 1.328, respectively. It is well known that when the JPDF of composite system suit the independent principle of multiplying, the entropy of composite systems is equal to the sum of the entropy of each subsystem. After that, we can numerically calculate the entropy of composite systems from the obtain JPDF ${p}_{{u}_{1},{u}_{2}}(y,z)$ as

12 $${S}_{{u}_{1},{u}_{2}}=-k\int {\int }_{W}{p}_{{u}_{1},{u}_{2}}(y,z)\,{ln}\,[{p}_{{u}_{1},{u}_{2}}(y,z)]dydz$$

From the currency exchange databases, we can obtain that the joint entropy of EUR/USD and GBP/USD equals 2.616, the joint entropy of EUR/USD and AUD/USD equals 2.619. It is important to note that the entropy of composite systems having cross correlations is not equal to the sum of the entropy of each subsystem. Since the information of different currency exchange systems may overlap, the entropy of joint currency exchange systems is less than the sum of the entropy of each currency exchange system. In addition, as the cross correlation of EUR/USD joint GBP/USD is larger than EUR/USD joint AUD/USD, the overlap information of EUR/USD joint GBP/USD may be larger than EUR/USD joint AUD/USD. As a result, the entropy of EUR/USD joint GBP/USD may be less than EUR/USD joint AUD/USD. With the same method, we can analyse other cross correlation properties of composite systems base on the obtained JPDF. It is also important to note that the results presented here do not need to know the form of interaction of composite systems. Furthermore, this method we proposed may play an important role in the more precise calculation of cross correlation properties in composite systems and go for the broad research of composite systems, including financial, artificial, social and natural^18–22^.

In summary, we present a unique way to acquire the cross correlation properties of composite systems. We can describe the JPDF only with six parameters in all different cases. It is found that the JPDFs between joint typical currency exchange systems do not suit the independent principle of multiplying of each system. It is also shown clearly that the entropy of composite systems having the cross correlation is not equal to the sum of the entropy of each system. In addition, we also found that, when the cross correlation of composite systems is larger, the entropy of composite systems is less. We must also note that the results presented here do not need to know the form of interaction of composite systems. It is anticipated that the further research in this direction may be generate more innovative ideas, create new light on the research of the composite systems which having the cross correlation.

## References

[1] Pressé S, Ghosh K, Lee J, Dill KA (2013). Principles of maximum entropy and maximum caliber in statistical physics. Rev. Mod. Phys..

[2] Tsallis C (1988). Possible Generalization of Boltzmann-Gibbs Statistics. J. Stat. Phys..

[3] Tsallis, C. In *Introduction to Nonextensive Statistical Mechanics: Approaching a Complex World*; Springer, New York, (2009**)**.

[4] Meir Z (2016). Dynamics of a Ground-State Cooled Ion Colliding with Ultracold Atoms. Phys. Rev. Lett..

[5] Pressé S, Ghosh K, Lee J, Dill KA (2013). Nonadditive Entropies Yield Probability Distributions with Biases not Warranted by the Data. Phys. Rev. Lett..

[6] Abe S (2014). Conditional maximum-entropy method for selecting prior distributions in Bayesian statistics. EPL.

[7] Huang Z (2014). Conditional statistical properties of the complex systems having long-duration memory. Physica A.

[8] Khinchin, A. Y. In *Mathematical Foundations of Statistical Mechanics*; Dover, New York, **(**1949**)**.

[9] Shlesinger, M. F. Zaslavsky, G. M. & Frisch, U. In *Lévy Flights and Related Topics in Physics*; Springer, Berlin, (1995).

[10] Mantegna RN, Stanley HE (1996). Turbulence and Financial Markets. Nature.

[11] Kutner, R. Pekalski, A. & Sznajd-Weron, K. In *Anomalous Diffusion: From Basics to Applications*; Springer-Verlag, Berlin, (1999).

[12] Metzler R, Klafter J (2000). The random walk’s guideto anomalous diffusion:a fractional dynamics approach. Phys. Rep..

[13] Caruso F (2007). Analysis of self-organized criticality in the Olami-Feder-Christensen model and in real earthquakes. Phys. Rev. E.

[14] Cai S (2007). Scale invariance of human electroencephalogram signals in sleep. Phys. Rev. E.

[15] DeVoe RG (2009). Power-Law Distributions for a Trapped Ion Interacting with a Classical Buffer Gas. Phys. Rev. Lett..

[16] Combe G (2015). Experimental Validation of a Nonextensive Scaling Law in Confined Granular Media. Phys. Rev. Lett..

[17] Tsallis C (1995). Statistical-Mechanical Foundation of the Ubiquity of Lévy Distributions in Nature. Phys. Rev. Lett..

[18] Mantegna RN, Stanley HE (1995). Scaling Behaviour in the Dynamics of an Economic Index. Nature.

[19] Mantegna, R. N. & Stanley, H. E. In *Introduction to Econophysics*; Cambridge University Press, Cambridge, (2000).

[20] Axtell RL (2001). Zipf distribution of U.S. firm sizes. Science.

[21] Borland L (2002). Option Pricing Formulas Based on a Non-Gaussian Stock Price Model. Phys. Rev. Lett..

[22] Clara-Rahola J (2017). Diffusive and Arrestedlike Dynamics in Currency Exchange Markets. Phys. Rev. Lett..

[23] Shannon CE (1948). A Mathematical Theory of Communication. The Bell System Technical Journal..

